# The Role of Laparoscopic Surgery in the Treatment of Advanced Uterine Prolapse: A Systematic Review of the Literature

**DOI:** 10.7759/cureus.18281

**Published:** 2021-09-25

**Authors:** Argirios Rountis, Dimitris Zacharakis, Stavros Athanasiou, Nikolaos Kathopoulis, Themos Grigoriadis

**Affiliations:** 1 Obstetrics and Gynecology Unit, First Department of Obstetrics and Gynecology, National and Kapodistrian University of Athens, Alexandra Hospital, Athens, GRC; 2 Urogynecology Unit, First Department of Obstetrics and Gynecology, National and Kapodistrian University of Athens, Alexandra Hospital, Athens, GRC; 3 Minimally Invasive Surgery Unit, First Department of Obstetrics and Gynecology, National and Kapodistrian University of Athens, Athens, GRC; 4 Urogynecology Unit, First Department of Obstetrics and Gynecology, National and Kapodistrian University of Athens, Athens, GRC

**Keywords:** severe pelvic organ prolapse, advanced pelvic organ prolapse, uterine prolapse, laparoscopy, laparoscopic surgery

## Abstract

The aim of this review is to investigate and compare all laparoscopic techniques that can be used in the surgical repair of advanced uterine prolapse. A systematic search of the PubMed, Scopus, Cochrane CENTRAL, and Clinicaltrials.gov databases was performed for articles published up to December 2020, reporting data on the treatment of severe uterine prolapse using laparoscopic procedures. Only studies in the English language, with a patient sample of ≥20 and a follow-up time of ≥12 months were included. The final synthesis of this review consisted of six studies. The main laparoscopic procedures reported were vaginally assisted laparoscopic sacrocolpopexy, vaginally assisted laparoscopic uterine sacropexy, laparoscopic sacrocolpopexy with laparoscopic supracervical hysterectomy, laparoscopic inguinal ligament suspension with uterine preservation, and laparoscopic uterosacral ligament suspension combined with trachelectomy. All procedures involved mesh placement, except for laparoscopic uterosacral ligament suspension. All procedures reported anatomical cure rates > 90%. Vaginally assisted laparoscopic sacrocolpopexy had the largest amount of intraoperative blood loss whilst vaginally assisted laparoscopic uterine sacropexy was associated with bladder injuries intraoperatively. All vaginally assisted procedures reported cases of mesh extrusion postoperatively. Laparoscopic inguinal ligament suspension was the operation with the longest mean operative and hospitalization time. Conversions were not reported. The present study shows that minimally invasive surgery can be used efficiently as an alternative to open surgery in the treatment of severe uterine prolapse.

## Introduction and background

Pelvic organ prolapse (POP) is defined as the descent of one or more of the anterior vaginal wall, posterior vaginal wall, uterus (cervix), or the apex of the vagina (vaginal vault or cuff scar after hysterectomy) [1]. Based on the pelvic organ prolapse quantification (POP-Q) system, there are five stages of prolapse severity, with stages III-IV representing the most advanced/severe cases of POP [2]. POP-related symptoms include bulge symptoms, lower urinary tract symptoms, bowel symptoms, and symptoms related to sexual dysfunction [3-4]. Risk factors for POP include increased age, obesity, multiparity, history of instrumented vaginal delivery, ≥ 10 years since menopause, and a family history of POP [5-6].

The exact prevalence of anatomically advanced prolapse in the general population is difficult to establish and most data regarding the distribution of pelvic organ support in women are based on gynecologic clinic populations. Indeed, in three different observational studies reporting on the prevalence of severe POP in women undergoing annual pelvic examination, the percentage of women with POP stage ≥ III was estimated at 0.6-2.6%, depending on the study [7-9].

Advanced POP is generally treated with surgery, especially if bothersome symptoms are severe and conservative treatment has failed [10]. However, the surgical repair of severe POP is considered a surgical challenge, as these patients often suffer from multicompartmental defects, which ideally should all be identified and addressed at primary surgery [11-12]. Additionally, it has been shown to be associated with a higher risk of developing recurrent prolapse after surgery [11,13].

Surgical treatment of advanced prolapse may be achieved through an abdominal or a transvaginal approach. Abdominal procedures include open, laparoscopic, or robotic-assisted routes. Fixation of the vaginal apex may be performed at the promontory, the sacrospinous ligament, the iliopectineal ligaments, the uterosacral ligaments, the inguinal ligament and include sacrocolpopexy, sacrouteropexy, sacrospinous fixation, pectopexy, uterosacral ligament suspension, and inguinal ligament suspension [14-16]. Modifications using a combination of both laparoscopic and transvaginal techniques have also been described [17-19].

The objective of this review is to investigate the laparoscopic techniques used in the surgical repair of advanced uterine prolapse, to compare them regarding their outcomes, and to discuss their indications in the treatment of severe POP based on the most recent data in the literature.

## Review

Materials and methods

 The present study was conducted according to the Preferred Reporting Items for Systematic Reviews and Meta-Analyses (PRISMA) guidelines [20].

Literature search

 A systematic search of the PubMed (1966-2020), Scopus (2004-2020), Cochrane CENTRAL Register of Controlled Trials (1996-2020), and Clinicaltrials.gov (2008-2020) databases was performed for articles published up to December 2020 using the combination of keywords "severe pelvic organ prolapse" OR "advanced pelvic organ prolapse," AND "laparoscopic surgery."

Eligibility criteria

All English-language studies, enrolling ≥ 20 patients, with follow-up ≥ 12 months and reporting on the treatment of advanced uterine prolapse (stage ≥ III by the POP-Q system) with laparoscopic techniques were included in this review. Randomized controlled trials, clinical trials, cohort studies, and case series were also included in this review while editorials, letters to the editor, case reports, reviews, and meta-analyses were excluded.

Screening process

The titles and abstracts of the articles that resulted from the literature search were screened to determine which studies were relevant to our object. Once the duplicates were recognized and removed, all relevant articles were then retrieved in full text and reviewed by two separate authors for inclusion or exclusion in accordance with our eligibility criteria. The reference lists of the articles that were retrieved in full text and included in this review were additionally searched for relevant articles in the field that may have been missed by the digital search, and any eligible articles identified in this way were also included in this review. Any discrepancies on selection were resolved by the consensus of all authors.

Data extraction

Our extracted data included patient demographics such as age, body mass index (BMI), parity, menopausal status, and surgical history, as well as POP characteristics (type, stage, and associated symptoms). Moreover, operation parameters, including the type of operation, operative time, estimated blood loss, intra/postoperative complications, and conversions, were also assessed. Hospital stay and concurrent procedures were additionally appraised. Finally, we evaluated the treatment outcome of each surgical operation by assessing four different parameters: i. Anatomical cure rate, ii. Presence of recurrent prolapse after surgery, iii. Patient satisfaction, iv. Post-operative symptoms/findings.

Results

**Figure 1:**
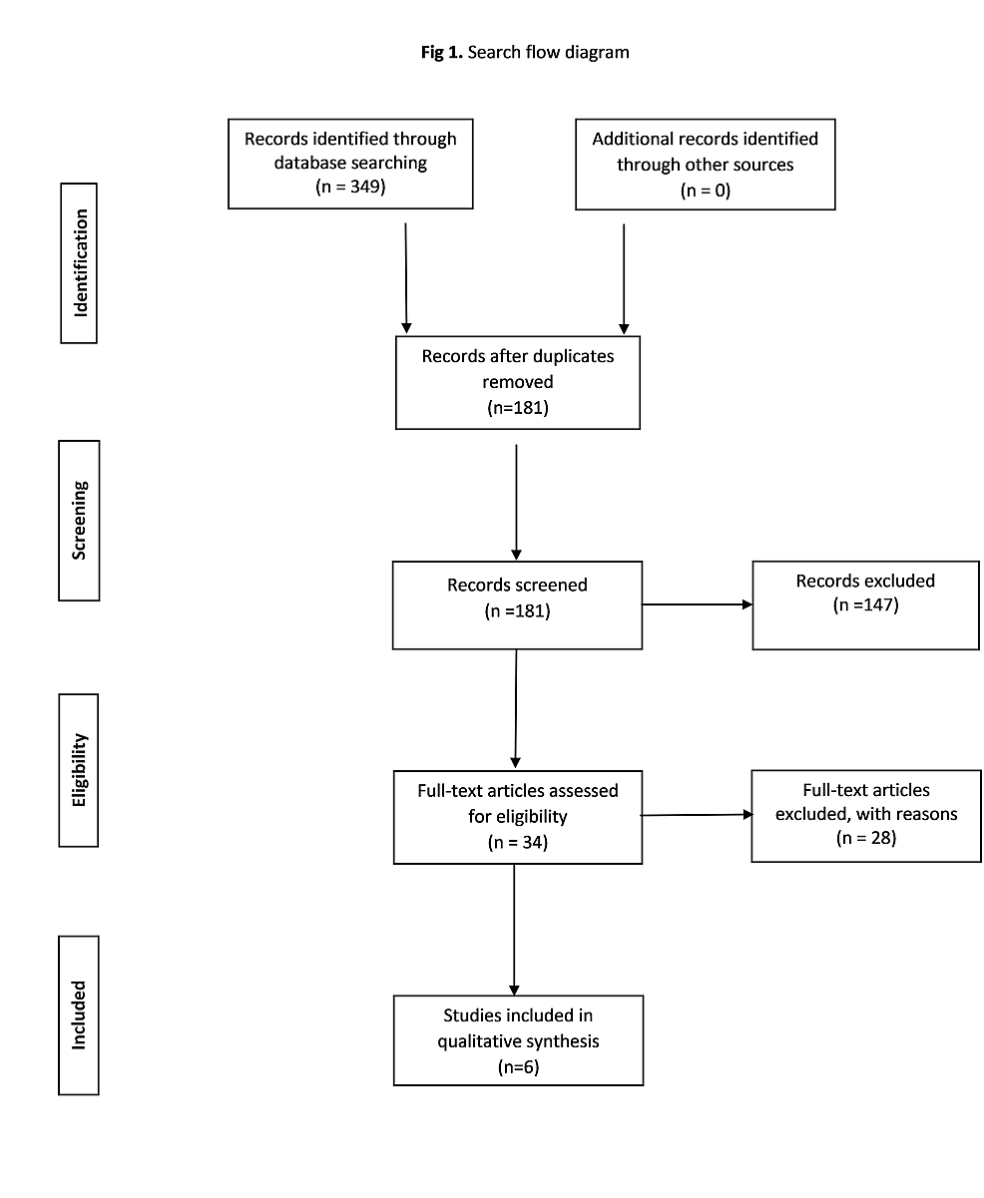
PRISMA search flow diagram PRISMA: Preferred Reporting Items for Systematic Reviews and Meta-Analyses

The database search using the aforementioned combination of keywords identified 349 records from PubMed (n = 269), Scopus (n = 63), Cochrane Library (n = 17), and ClinicalTrials.gov (n = 0). After the removal of the duplicates, the remaining 181 records were screened for eligibility based on the title and the abstract of the article. This searching strategy resulted in 34 English-language articles that were retrieved in full text. Subsequently, those articles were reviewed by two different authors for inclusion or exclusion according to our predetermined eligibility criteria. After the exclusion of 28 articles that involved hysterectomized women, patients with POP stage <ΙΙΙ, follow-up time<12 months, or patient sample size<20, six eligible articles emerged from this process and were included in our review. Figure 1 summarizes our data search strategy.

Quality Assessment

The quality of the included RCTs was evaluated using the modified Jadad score [21]. The quality of the non-randomized studies was assessed according to Methodological Items for Non-Randomized Studies (MINORS) [22]. Two authors independently performed the procedure. The Jadad scale for the included RCT scored 5 (max 5) and the MINORS scale provided a mean score of 12.7 for the four included non-comparative studies (range 12-14, max16) and a score of 22 for the included comparative study (max 24).

Characteristics of the Included Studies

The qualitative synthesis of this review consisted of one randomized controlled trial (RCT) [23], four prospective studies [17-19,24], and one retrospective study [25] and included a total of 359 patients. Vaginally assisted laparoscopic sacrocolpopexy (VALS) was the most common procedure performed for the treatment of severe uterovaginal prolapse (two studies) [17,19] followed by laparoscopic sacrocolpopexy (LSH) combined with laparoscopic supracervical hysterectomy (one study) [23]. Uterine-preserving techniques included laparoscopic inguinal ligament suspension (LILS) with mesh (1 study) [25], combined trachelectomy with laparoscopic uterosacral ligament suspension (LULS) (1 study) [24], and vaginally-assisted laparoscopic uterine sacropexy (VALUES) (1 study) [18]. Five studies of this review reported on procedures using meshes, whereas, in only one study, native tissue was used during pelvic floor repair [24]. Table 1 presents the characteristics of the included studies.

**Table 1:** Characteristics of the included studies PS: Prospective study, RS: Retrospective study, RCT: Randomized control trial, LSC: Laparoscopic sacrocolpopexy, LSH: Laparoscopic supracervical hysterectomy, LILS: Laparoscopic inguinal ligament suspension, LULS: Laparoscopic uterosacral ligament suspension, VALS: Vaginally assisted laparoscopic sacrocolpopexy, VALUES: Vaginally-assisted laparoscopic uterine sacropexy

Author	Study design	Number of patients	Main type of operation	Mean/median follow-up (months)
Athanasiou et al., 2012 [17]	PS	27	VALS with mesh	2 follow-up visits at 2 and 12 months
Fayyad et al., 2013 [18]	PS	70	VALUES with mesh	2 follow-up examinations at 3 and 12 months
Sun et al., 2015 [24]	PS	49	Trachelectomy and LULS without mesh	54
Athanasiou et al., 2018 [19]	PS	94	VALS with mesh	84
Morciano et al., 2018 [23]	RCT	84 (42 per group)	LSH plus a “two-meshes” LSC with different mesh fixation between the two groups	A 12-month follow-up
			Group1: extracorporeal interrupted 3-0 delayed absorbable sutures	
			Group2: running locked 3-0 delayed absorbable suture	
Li et al., 2018 [25]	RS	35	LILS using a mesh with uterine preservation	15.3±5.1

Patient Demographics, POP Characteristics, and Preoperative Symptoms

Patient demographics are presented in detail in Table 2. All studies included in this review involved patients with uterovaginal prolapse stage ≥ III by the POP-Q system with or without concurrent cystocele/rectocele. Voiding symptoms and urgency were reported as preoperative symptoms in one study in our review [18]. Additionally, two studies involved a total of 42 patients (11.7%) with concomitant urinary stress incontinence (USI) preoperatively [18-19], and one study reported eight patients (2.2%) with urge incontinence (UI) prior to surgery [18]. In four studies, data regarding preoperative symptoms were not available [17,23-25]. Table 3 lists POP characteristics and patient preoperative symptoms.

**Table 2:** Patient demographics N/A: Not available, BMΙ: Body mass index

Author	Mean/median age (years)		Mean BMI (kg/m^2^)		Mean parity		Menopause
Athanasiou et al., 2012 [17]	57.2		25.4		2.3		20/27
Fayyad et al., 2013 [18]	61		29		2		41/70
Sun et al., 2015 [24]	37.1		21.8 ± 1.9		1.1		N/A
Athanasiou et al., 2018 [19]	56		24.8		1-2: 71/94	>2: 23/94	N/A
Morciano et al., 2018 [23]	Group I:66	Group II:64	Group I:24	Group II:25	Nulliparous: Group I:3/42	Group II:4/42	N/A
Li et al., 2018 [25]	48		23.1±3.2		1.4 ± 0.6		N/A

**Table 3:** POP type, stage, and associated symptoms POP: Pelvic organ prolapse, USI: Urinary stress incontinence, UI: Urge incontinence, N/A: Not available

Author	POP type and stage							Symptoms	
Athanasiou et al., 2012 [17]	Uterovaginal prolapse			stage III 20/27			stage IV 7/27	N/A	
Fayyad et al., 2013 [18]	Uterine prolapse			stage III 43/70			stage IV 27/70	Urgency 37/70	Dribble 18/70
								UI 8/70	Frequency 31/70
								USI 5/70	Poor stream 25/70
								Strain to empty the bladder 26/70	
Sun et al., 2015 [24]	Uterine prolapse stage ≥III-IV 49/49							N/A	
	Cystocele	Rectocele							
	stage I 31/49	stage I 26/49							
	stage II 15/49	stage II 18/49							
	stage III 3/49	stage III 5/49							
Athanasiou et al., 2018 [19]	Apical prolapse:	stage III 52/94				stage IV 42/94		USI 37/94	Detrusor overactivity 14/94
	Cystocele				Rectocele				
	stage I 0/94				stage I 15/94				
	stage II 6/94				stage II 32/94				
	stage III 50/94				stage III 12/94				
	stage IV 38/94				stage IV 35/94				
Morciano et al., 2018 [23]	Uterine prolapse and cystocele stage III-IV 84/84							N/A	
	Median POP-Q stage III in both groups.								
Li et al., 2018 [25]	Symptomatic POP:		stage III 26/35			stage IV 9/35		N/A	

Previous Pelvic/Abdominal Operations

Two studies in our review involved patients that had undergone pelvic/abdominal operations in the past [18,23]. More specifically, in the Morciano et al. RCT, 18/84 (21.4%) patients had been subjected to a cesarian section in the past, whilst 11/84 (13.1%) patients reported a history of other previous abdominal procedures [23]. Additionally, in the Fayyad et al. study, 6/70 (8.6%) patients had a previous operation for anterior vaginal wall repair [18]. Three studies reported no previous pelvic or abdominal operations [17,19,25], and in one study, data regarding the patient’s surgical history were not available [24]. Previous pelvic/abdominal operations are shown in Table 4.

**Table 4:** Previous pelvic/abdominal operations Ν/Α: Νot available, POP: Pelvic organ prolapse

Author	Previous operations	
Athanasiou et al., 2012 [17]	No previous pelvic or abdominal operations	
Fayyad et al., 2013 [18]	Anterior vaginal wall repair 6/70	
Sun et al., 2015 [24]	N/A	
Athanasiou et al., 2018 [19]	No previous pelvic or abdominal operations	
Morciano et al., 2018 [23]	Cesarean section: Group I: 8/42	Group II: 10/42
	Abdominal surgery: Group I: 6/42	Group II: 5/42
	No previous POP operations in neither group	
Li et al., 2018 [25]	No previous POP operations	

Operation-Related Data

The mean operative time varied depending on the procedure within a range of 51-164 min. More specifically, combined trachelectomy with LULS had the shortest mean duration, which was 51.0 ± 8.4 min [24], whilst uterine LILS with mesh was the procedure with the longest mean operative time, estimated at 163.8 ± 41.3 min [25]. Furthermore, trachelectomy with LULS was the operation with the least intraoperative bleeding (32.0 ± 17.5 ml) [24]. On the contrary, vaginally assisted laparoscopic sacrocolpopexy had the largest amount of intraoperative blood loss (310 ml) [17].

None of the studies included in this review reported conversions to open surgery. Hospitalization time varied within a range of 36 hours-five days and was associated with the type of procedure. Patients that underwent VALUES with mesh reported the shortest hospitalization time (36 hours) [18], whilst patients that were subjected to uterine LILS with mesh required the longest hospital stay (five days) [25]. Table 5 summarizes all the operative data from the included studies.

Complications

In our review, we separated surgical-related complications into two main categories: intraoperative complications and postoperative complications.

Five out of the six included studies reported no intraoperative complications [17,19,23-25]. In the Fayyad et al. study, two inadvertent bladder injuries (2, 9%) occurred during VALUES, which were repaired successfully at the time of surgery [18].

Neither of the included studies reported mesh erosion postoperatively. On the contrary, mesh exposure was reported in two studies [18-19]. More specifically, Fayyad et al. [18] reported one case of post-menopausal bleeding combined with mesh exposure after VALUES, whilst Athanasiou et al. reported two cases of mesh extrusion after VALS [19]. Based on the aforementioned studies, the mesh exposure rates after VALUES and VALS in our review were estimated at 1.4% and 2.1%, respectively.

Postoperative fever was reported in Morciano et al. RCT [23] after LSH plus LSC in 3/84 (3.6%) patients, whilst pelvic hematoma occurred in 2/70 (2.8%) patients in Fayyad et al. study [18] after VALUES. Finally, in the Athanasiou et al. study [17], one patient (3.7%) complained about the presence of a prolene suture visible at the vaginal vault two months after VALS, which was ultimately removed under local anesthesia. All intra- and postoperative complications for each study can be found in detail in Table 5.

**Table 5:** Operation-related data and complications N/A: Not available

Author	Mean/ median operative time (min)	Average blood loss (ml)	Intra/post-operative complications	Conversion	Hospital stay
Athanasiou et al., 2012 [17]	Vaginal hysterectomy and mesh placement 74 Laparoscopic suspension 64	310	No intra-operative complications. Presence of the prolene suture visible at the vaginal vault 2 months postoperatively 1/27	0/27	2.8 days
Fayyad et al., 2013 [18]	122	100	Bladder injury 2/70; Pelvic hematoma 2/70; Post-menopausal bleeding plus mesh exposure 1/70	N/A	36 hours
Sun et al., 2015 [24]	51.0 ± 8.4	32.0 ± 17.5	No intra or postoperative complications	N/A	N/A
Athanasiou et al., 2018 [19]	N/A	N/A	No intraoperative complications. Mesh extrusion 2/94	0/94	N/A
Morciano et al., 2018 [23]	Operative time: Group I: 138, Group II: 121; Mesh fixation: Group I: 39, Group II: 24	Group I: 60, Group II: 50	No intra-operative complications in either group. Postoperative fever: Group I: 1/42, Group II: 2/42	N/A	N/A
Li et al., 2018 [25]	Operative time 163.8 ± 41.3; Mesh fixation 85.5 ± 18.6	48.6 ± 60.5	No serious intra/postoperative complications	0/35	5 days

Concurrent Procedures

Three studies reported hysterectomy (total or subtotal) as one of the main concomitant procedures [17,19,23]. Morciano et al. [23] performed laparoscopic supracervical hysterectomy in all their patients prior to LSC, whilst in both Athanasiou et al. studies [17,19] all patients underwent vaginal hysterectomy during the first step of the VALS operation. Unilateral/bilateral salpingo-oophorectomy was also performed when indicated during hysterectomy, with laparoscopy being the preferred approach for the procedure [17,19].

Concurrent pelvic floor repair operations were also reported in our review. More specifically, in three different studies [17-18,24], a total of 74 patients (21.2%) underwent anterior colporrhaphy for concomitant cystocele, whilst three studies reported a total of 74 rectocele cases (21.2%) that were subjected to a concomitant posterior colporrhaphy/perineorrhaphy for their treatment [17,19,24]. Moreover, four studies involved a total of 51 patients (14.6%) that underwent a sling placement procedure for USI symptoms [17-19,24]. Finally, other concomitant operations reported were intrauterine devices removal, ovarian cyst resection, and diagnostic curettage [24]. Table 6 summarizes all concurrent procedures for each study.

**Table 6:** Concurrent procedures TVT-O: Tension-free vaginal tape-obturator (procedure), N/A: Not available

Author	Concurrent procedures	
Athanasiou et al., 2012 [17]	Vaginal hysterectomy 27/27, Posterior colporrhaphy/perineorrhaphy 5/27	
	Transobturator mid-urethral sling 9/27, Anterior colporrhaphy 1/27	
	Laparoscopic salpingo-oophorectomy 23/27	
Fayyad et al., 2013 [18]	Anterior colporrhaphy 70/70	
Sun et al., 2015 [24]	Anterior colporrhaphy 3/49	Posterior colporrhaphy 5/49
	TVT-O procedure 2/49	Tension-free vaginal tape procedure 3/70
	Diagnostic curettage 1/49	Ovarian cyst resection 2/49
	Intrauterine devices removal 3/49	
Athanasiou et al., 2018 [19]	Vaginal hysterectomy 94/94, Posterior colporrhaphy/perineoplasty 64/94	
	Bilateral salpingo-oophorectomy 54/94, TVT/TVT-O 37/94	
Morciano et al., 2018 [23]	Laparoscopic supracervical hysterectomy 84/84	
Li et al., 2018 [25]	N/A	

Treatment Outcomes

The treatment outcomes of each surgical operation were evaluated by assessing four different parameters: i. Anatomical cure rate, ii. Presence of recurrent prolapse after surgery, iii. Patient satisfaction, iv. Postoperative symptoms/findings. Table 7 lists, in detail, the aforementioned parameters for each study.

**Table 7:** Treatment outcomes N/A: Not available, UI: Urge incontinence, USI: Urinary stress incontinence, POP: Pelvic organ prolapse, PGI-I: Patient Global Impression of Improvement

Author	Anatomical cure rate	Recurrence		Patient satisfaction	Post-operative symptoms/findings			
Athanasiou et al., 2012 [17]	100%	0/27		PGI-I scale	Urinary voiding difficulties 1/27			
				"very much better" 19/27				
					De novo constipation 3/27			
				"much better" 6/27				
				"better" 2/27				
Fayyad et al., 2013 [18]	91.4%	Anterior vaginal wall prolapse stage ≥II 3/70; Uterine prolapse stage IV 3/70		PGI-I scale	Urgency 6/70			
				"very much better" 30/70				
					UI	Total 3/70	De novo 1/70	
					Frequency	Total 13/70	De novo 1/70	
				"much better" 30/70	Dribble	Total 8/70	De novo 2/70	
					Poor stream	Total 12/70	De novo 0/70	
				"little better" 5/70	Strain	Total 13/70	De novo 2/70	
				"same" 5/70	USI	Total 10/70	De novo 6/70	
					De novo dyspareunia 1/70			
Sun et al., 2015 [24]	100%	0/49		100%	N/A			
Athanasiou et al., 2018 [19]	95.7%	Posterior compartment prolapse 3/94		PGI-I scale	Vaginal bulge symptoms without anatomic recurrence 1/94			
				‘"very much better" 75/94				
				"much better" 12/94				
				"better" 7/94				
Morciano et al., 2018 [23]	97.6% in each group	Anatomic failure		N/A	N/A			
		Group I: 1/42	Group II: 1/42					
Li et al., 2018 [25]	94.3% at 12-month follow-up	POP stage III 2/35		N/A	N/A			

Anatomical cure rate: The anatomical cure rates between the different surgical approaches regarding the treatment of severe uterovaginal prolapse were similar in our review, with no significant differences being reported. All procedures resulted in an anatomical cure rate of >90%, within a range of 91.4-100%.

More specifically, in the Morciano et al. RCT [23], LSH plus LSC with mesh resulted in an anatomical cure rate of 97.6% in each treatment group, whereas in the Athanasiou et al. prospective studies, the VALS anatomical cure rate was estimated at 100% [17] and 95.7% [19], respectively, differences that can be attributed to the longest follow-up and largest sample size of the second study.

In terms of uterine preservation, LILS of the uterus showed an anatomical cure rate of 94.3% in the Li et al. study [25], whereas the VALUES procedure resulted in a 91.4% success rate in the Fayyad et al. study [18]. Finally, Sun et al. [24] reported a 100% anatomical cure rate for combined LULS with trachelectomy in the treatment of advanced uterovaginal prolapse.

Based on the aforementioned studies, the anatomical cure rate for each procedure was 97.6% for LSC plus LSH, 95.7-100% for VALS, 94.3 for LILS of the uterus, 100% for combined LULS with trachelectomy, and 91.4% for VALUES.

Recurrent prolapse: Four out of the six included studies in our review reported postoperative recurrences [18-19,23,25] More specifically, one study reported recurrences after LSC plus LSH [23], one study after VALS [19], one study after VALUES [18], and one study after LILS with uterine preservation [25]. Two studies reported no recurrences during the follow-up period [17,24].

As far as the LSC plus LSH procedure is concerned, in the Morciano et al. RCT where anatomic failure was defined as prolapse stage ≥ II in any site, the recurrence rate was estimated at 2.4% in each LSC treatment group [23]. Furthermore, in the Athanasiou et al. study (2018), failures after VALS (4.3%) included one case of anatomical recurrence (1.1%), one woman reporting vaginal bulge symptoms postoperatively (1.1%), and two cases of reoperation (2.1%) [19]. Additionally, Fayyad et al. reported as recurrences after VALUES three cases of anterior vaginal wall prolapse stage ≥II (4.3%) and three cases of uterine prolapse stage IV (4.3%) [18]. Finally, in the Li et al. study, two cases of prolapse stage ≥III (5.7%) were reported as recurrences after LILS with uterine preservation [25]. Based on the included studies of this review, recurrence rates for LSC plus LSH, VALS, VALUES, LILS of the uterus, and LULS with trachelectomy were 2.4%, 0-4.3%, 8.6%, 5.7%, and 0%, respectively.

Patient satisfaction: Patient satisfaction was reported in four out of the six included studies [17-19,24]. In three of them, it was evaluated by using the Patient Global Impression of Improvement (PGI-I) scale [17-19], whereas in one study, the question: “How satisfied are you with the results of your surgery?” was used to determine patient satisfaction after surgery [24].

More specifically, in the Athanasiou et al. study in 2012 [17], 92.5% of patients reported being ‘’very much better’’ or ‘’much better’’ after the VALS operation, with the same percentage giving the same answers in the study that followed in 2018 [19]. Moreover, in the Fayyad et al. study [18], 85.7% of the patients reported being ‘’very much better’’ or ‘’much better’’ after VALUES for advanced uterine prolapse, whereas, in the Sun et al. study [24], combined LULS and trachelectomy achieved a 100% satisfaction rate in treating severe uterine prolapse. Finally, data on patient satisfaction were not available in the Morciano et al. and Li et al. studies [23,25]. Based on the aforementioned studies, patient satisfaction for each operation was estimated at 92.5% for VALS, 90.5% for LILS of the uterus, 100% for combined LULS with trachelectomy, and 85.7% for VALUES.

Postoperative symptoms/findings: Postoperative patient symptoms were reported in three out of the six included studies [17-19]; in the other three studies, data regarding patient symptoms after surgery were not available [23-25].

More specifically, Athanasiou et al. [17] reported urinary voiding difficulties in one patient (3.7%) and de novo constipation in three patients (11.1%) after VALS, whereas, in their study that followed in 2018 [19], only one patient (1.1%) complained about vaginal bulge symptoms without anatomic recurrence. Moreover, the most common symptoms after VALUES in the Fayyad et al. study were voiding difficulties (strain - 18.6%, poor stream - 17.1%, dribble - 11.4%) and urinary frequency disorders (18.6%) with only 4.3% of the patients having undergone a concurrent tape procedure for USI at primary surgery. Other symptoms included urgency (8.6%), USI (14.3%), UI (4.3%) and dyspareunia (1.4%) [18].

Discussion

The present study indicates that laparoscopic surgery, although more technically demanding and time-consuming, is associated with less intraoperative bleeding [23-25], shorter hospitalization time [18], and similar anatomical outcomes compared to open surgery regarding the treatment of advanced apical prolapse, which is in accordance with current literature [26-27]. However, surgeons should be aware that adequate surgical skills and a good learning curve of the operation that they perform is needed in order to ensure a satisfactory anatomic result and safety for their patients [28].

Age, BMI, menopausal status, and parity should be taken into consideration before proceeding in the surgical treatment of severe POP. In advanced-age, obese, postmenopausal women with severe uterine prolapse, removal of the uterus is preferred to hysteropexy due to the fact that these women are at increased risk of endometrial cancer development if the uterus remains [29]. However, hysterectomy alone is not an adequate treatment and an apical suspension procedure should be performed at the same time in order to reduce the risk of recurrent POP [10,30]. 

VALS can be effectively performed in those women in order to correct not only their severe uterovaginal prolapse but also the concurrent multicompartmental prolapse that these women may have, resulting in anatomical cure rates of 95.7-100% in our review [17,19]. Although this technique resulted in the highest mesh extrusion rate in our review (2.1%), this rate is considered to be generally low, taking into account that the reported incidence of the mesh-related complications, including mesh extrusion, in patients undergoing LSC with the use of polypropylene mesh, is up to 3.4%, and that this rate is significantly increased in cases of vaginal insertion of the mesh [31-32].

Alternatively, laparoscopic hysterectomy combined with LSC can be performed for POP repair in this patient group with similar anatomic outcomes (97.6%) but longer operative times that can be attributed to the laparoscopic route of hysterectomy during this procedure. As far as operative time is concerned, the Morciano et al. study showed that a single running locked suture is preferred to multiple interrupted stitches during mesh fixation, as it can significantly reduce the mesh fixation time (24 vs. 39 min; p < 0.01) and total operative time (121 vs. 138 min; p < 0.05) of the aforementioned procedure [23].

On the other hand, in young nulliparous women with uterine prolapse, preservation of the uterus is equally important to anatomical success [33-34]. Moreover, hysterectomy is usually associated with vaginal shortening, which may result in sexual dysfunction [35]. Therefore, uterine preserving techniques, such as VALUES, LILS of the uterus, and trachelectomy with LULS, constitute good options for young, sexually active women who desire future childbearing [18,24-25].

More specifically, the VALUES procedure resulted in a 91.4% success rate in treating severe uterovaginal prolapse in the Fayyad et al. prospective study. The main advantage of VALUES included the extra-peritoneal attachment of the mesh to the cervix, which provided efficient support to the lower part of the cervix in cases of cervical elongation and allows easy cutting of the tail of the mesh from the sacrum should hysterectomy be required in the future. In terms of complications, VALUES was the only operation in our review with intraoperative complications, reporting two inadvertent bladder injuries during the procedure (2.9%), which were repaired successfully at the time of surgery. Postoperatively, the low incidence of mesh complications (1.4%) was explained by the rich blood supply to the vaginal part of the cervix where the mesh was attached and the use of light-weight type 1 polypropylene mesh, which reduces mesh complications [18].

Furthermore, LILS of the uterus can be effectively performed in women with severe prolapse that desires uterine preservation with anatomical cure rates estimated at 94.3% based on the data of our review. In the Li et al. study, no intraoperative complications were reported during LILS and none of the patients developed mesh-related complications postoperatively. The authors claimed that the absence of mesh complications postoperatively is a result of a technique of complete mesh peritonization that reduces the risk of mesh erosion into the rectum or ureter. However, LILS was associated with the longest hospitalization time in our review (5 days), a factor that should be taken into consideration when evaluating the surgical technique in terms of hospital cost [25].

Moreover, trachelectomy with LULS was the operation with the shortest mean surgical time in our review (51.0 ± 8.4 min), which additionally reported the least intraoperative bleeding (32.0 ± 17.5 ml) [24]. Although this uterine-sparing operation resulted in a 100% anatomical cure rate and reported no intra/postoperative complications, it is probably is not the best choice for women who desire childbirth due to the fact that trachelectomy is associated with preterm premature rupture of the membranes and preterm birth [36-37].

Women who present with severe uterovaginal prolapse are more likely to have defects that involve various levels of pelvic support [12]. Therefore, concomitant surgery should be performed during the primary procedure in order to address concurrent pelvic floor defects [13]. In the included studies of this review, the operation of choice for the treatment of concurrent cystocele was anterior colporrhaphy (21.2%) [17-18,24], whilst when a rectocele was present, it was treated with posterior colporrhaphy/perineorrhaphy (21.2%) [17,19,24]. Additionally, the presence of USI and other urinary symptoms was an indication for anti‐incontinence surgery, which included sling placements procedures (14.6%) [17-19,24].

Strengths and limitations

The strength of our study emerges from the meticulous analysis of all the parameters of the included studies and from the fact that all studies that were included in this review reported strictly on the treatment of uterine prolapse stage ≥III, without involving patients with lesser stages of POP that could alter the results of our study. Moreover, all included studies had a follow-up of at least 12 months and a population sample of > 20 patients. The limitations of our study are the small number of the included RCTs (1) and the fact that some data under examination are not available in some of the included studies. Finally, due to the restricted amount of existing evidence and the heterogeneous data of the included studies, no further statistical analysis was possible.

## Conclusions

It seems that the balance is in favor of laparoscopic surgery in terms of intraoperative blood loss and admission time, with similar anatomical outcomes compared to open surgery, as far as the treatment of severe uterine prolapse is concerned. However, more well-designed cohort studies are required in order to verify the results of this review and provide pelvic surgeons with more evidence for the management of this surgically demanding health problem.
